# Appropriate level of cuproptosis may be involved in alleviating pulmonary fibrosis

**DOI:** 10.3389/fimmu.2022.1039510

**Published:** 2022-12-19

**Authors:** Guoxing Li, Lihua Peng, Mingjun Wu, Yipin Zhao, Zhe Cheng, Gang Li

**Affiliations:** ^1^ Center for Novel Target and Therapeutic Intervention, Institute of Life Sciences, Chongqing Medical University, Chongqing, China; ^2^ Institute of Life Sciences, Chongqing Medical University, Chongqing, China; ^3^ Department of Cardiology, The First Affiliated Hospital of USTC, Division of Life Sciences and Medicine, University of Science and Technology of China, Hefei, China; ^4^ Department of Cardiology, Chongqing University Three Gorges Hospital, Chongqing, China; ^5^ Molecular Medicine Diagnostic and Testing Center, Chongqing Medical University, Chongqing, China

**Keywords:** copper, cuproptosis, pulmonary fibrosis, ScRNA-seq, TCA cycle

## Abstract

**Objective:**

Cuproptosis is a newly discovered form of programmed cell death that has not been studied in pulmonary fibrosis. The purpose of the present study was to explore the relationship between cuproptosis and pulmonary fibrosis.

**Methods:**

Single-cell sequencing (scRNA-seq) data for human and mouse pulmonary fibrosis were obtained online from Gene Expression Omnibus (GEO) database. First, fibroblast lineage was identified and extracted using the Seurat toolkit. The pathway was then evaluated *via* Gene Set Enrichment Analyses (GSEA), while transcription factor activity was analyzed using DoRothEA. Next, fibroblast differentiation trajectory was inferred *via* Monocle software and changes in gene expression patterns during fibroblast activation were explored through gene dynamics analysis. The trajectory was then divided into three cell states in pseudotime order and the expression level of genes related to cuproptosis promotion in each cell state was evaluated, in addition to genes related to copper export and buffering and key genes in cellular metabolic pathways.

**Results:**

In the mouse model of pulmonary fibrosis induced by bleomycin, the genes related to cuproptosis promotion, such as *Fdx1*, *Lias*, *Dld*, *Pdha1*, *Pdhb*, *Dlat*, and *Lipt1*, were gradually down-regulated in the process of fibroblast differentiation from resting fibroblast to myofibroblast. Consistently, the same results were obtained *via* analysis of scRNA-seq data for human pulmonary fibrosis. In addition, genes related to copper ion export and buffering gradually increased with the activation of fibroblasts. Metabolism reprogramming was also observed, while fibroblast activation and tricarboxylic acid(TCA) cycle and lipid metabolism were gradually down-regulated and mitochondrial metabolism was gradually up-regulated.

**Conclusion:**

The present study is the first to reveal a negative correlation between cuproptosis and fibrosis, suggesting that an appropriate cuproptosis level may be involved in inhibiting fibroblast activation. This may provide a new method for the treatment of pulmonary fibrosis.

## Introduction

Idiopathic pulmonary fibrosis (IPF) is a progressive interstitial lung disease. If it is untreated, the average life span after diagnosis is 3–5 years (1). IPF is a growing threat to public health worldwide with a great burden to human health and social economy (2). Pirfenidone and nintedanib are therapeutic drugs that are recommended by the treatment guidelines, although they have limited efficacy (3). Lung transplantation is the only current effective treatment, but it is limited by its high cost and rarity of donors (4). Part of IPF mechanism may involve persistent lung epithelial injury and myofibroblasts activation (5–7). Activation of myofibroblasts leads to excessive deposition of extracellular matrix and scar repair, thereby leading to tissue scar formation, alveolar structure deformation, and finally irreversible damage to lung function (8).

Excessive proliferation and activation of fibroblasts are characteristics of fibrosis, and promoting apoptosis seems to be an effective solution for fibrosis (9, 10). In addition, autophagy, as another form of programmed cell death, is involved in alleviating fibrosis (11, 12). However, cuproptosis, which is a newly discovered form of programmed cell death, has not been studied in fibroblasts (13). Whether normal copper ion concentration can induce cuproptosis in fibroblasts needs to be addressed, in addition to identifying any differences in cuproptosis level between resting fibroblasts and myofibroblasts. At present, little is known about the role of cuproptosis in pulmonary fibrosis.

Because of its unique single-cell resolution, single-cell sequencing plays an important role in exploring various disease mechanisms and may help to answer the above questions. The present study aimed to explore the mechanism of fibroblast activation in pulmonary fibrosis and the relationship between cuproptosis and fibrosis *via* single-cell sequencing analysis.

## Materials and methods

### Data processing

The datasets analysed in this study were obtained from the GEO database (https://www.ncbi.nlm.nih.gov/geo/) with accession number GSE132771. The following steps were performed on mouse and human data: a) preliminary data quality control was carried out based on percent.mito and nFeatures of each sample; b) samples were integrated using the merge function of Seurat (v4.0.5) (14) followed by data standardization; c) Harmony (v0.1.0) (15) was used for reclustering of fibroblast lineage; and d) the cells were manually annotated using existing knowledge or automatically annotated with SingleR (v1.8.1) (16) after clustering and UMAP or tSNE dimensional reduction. It is worth noting that there may have been errors in automatic annotation, so we excluded some non-fibroblasts according to marker genes after fibroblast lineage reclustering.

### Gene ontology enrichment analysis

GO analysis was performed *via* clusterprofileR package (v0.5.0) (17) with the following parameters: pAdjustMethod = “BH”, pvalueCutoff = 0.01, and qvalueCutoff = 0.05.

### Gene set enrichment analysis

The marker genes for each group were identified with a threshold of min.pct = 0.1 and logFC.threshold = 0 and then sorted according to logFC. The GSEABase (v1.56.0) package (http://www.bioconductor.org/packages/release/bioc/html/GSEABase.html ) was used to identify biological processes (BPs) of GO terms and Kyoto Encyclopedia of Genes and Genomes pathways.

### Assessment of transcription factor activity

Transcription factor activity was analyzed using DoRothEA (v1.8.1) (18) package, and all parameters were set to default values.

### Pseudotime trajectory inference

Monole2 (v2.22.0) (19) was used for cell pseudotime analysis. Cell progression genes were defined based on differentially expressed genes (DEGs) among Seurat clusters. DifferentialGeneTest function was used to explore gene dynamics during cell differentiation. Notably, due to the small number of cells with positive genes related to cuproptosis, it was difficult to show the changes in these genes using the plot_genes_in_pseudotime function. Therefore, the trajectory was divided into three segments according to the trajectory nodes. By evaluating the gene expression of cells in each segment, the changing trend in these genes with cell differentiation was revealed.

## Results

### Single-cell landscape of bleomycin-induced pulmonary fibrosis mouse model

**Figure 1:**
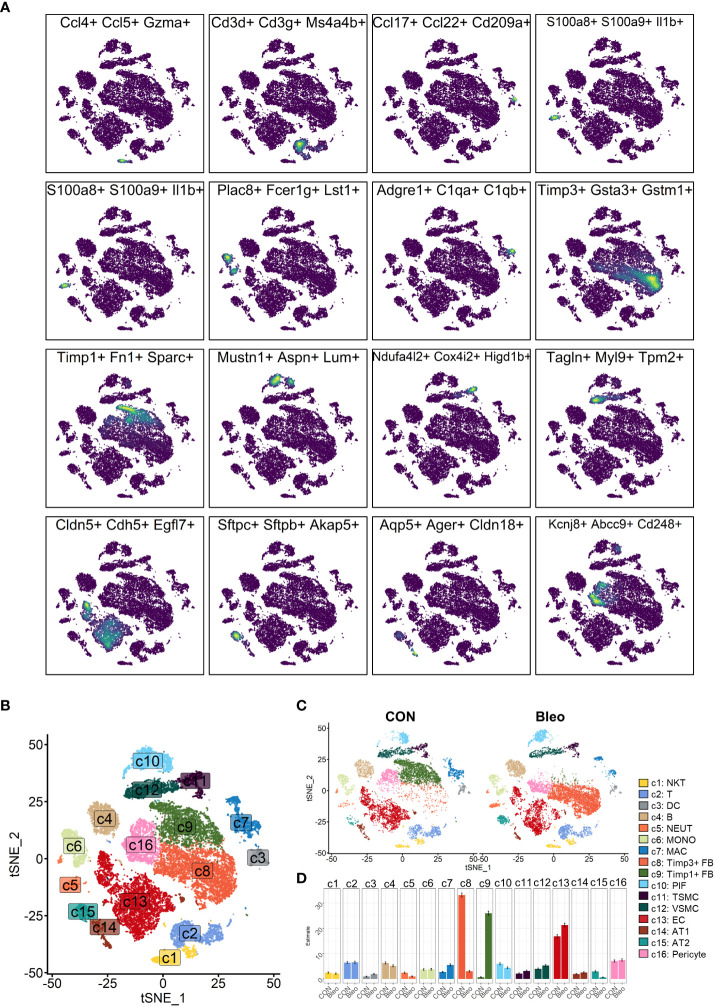
Clustering of 16 cell lineages. **(A)** t-SNE color-coded by annotated cell lineage. **(B)** t-SNE of two groups color-coded by cell lineage. **(C)** Proportion of different cell lineages in each group color-coded by cell lineage. **(D)** Representative markers of each cell lineage.

In the experiment of this data, a model of pulmonary fibrosis was induced *via* intratracheal instillation of bleomycin (Bleo) in two mice, with two control mice remaining untreated. After 14 days, lung tissue samples were harvested and single-cell sequencing was performed. In the present study, 24,471 high-quality cells were screened using the parameters of 200 < nFeatures < 4000 and percent.mito < 8%. Next, dimensionality reduction clustering was performed on the quality-controlled data, followed by data annotation based on the marker genes (**Figure 1A**). This process identified a total of 16 cell lineages (**Figure 1B**), including natural killer T cells(NKT), T cells(T), dendritic cells(DC), neutrophils(NEUT), monocytes(MONO), macrophages(MAC), TIMP3+ fibroblasts (TIMP3+ FB), TIMP1+ fibroblasts (TIMP1+ FB), pulmonary interstitial cells(PIF), trachea smooth muscle cells(TSMC), vascular smooth muscle cells(VSMC), endothelial cells(EC), type 1 alveolar cells(AT1), type 2 alveolar cells(AT2), and pericytes(Pericyte). The fibroblast compositions in the two groups were quite different. In fact, cells of other lineages did not show significant polarity, suggesting that the differences in fibroblasts were due to biological effects rather than batch effects (**Figure 1C**). The fibroblasts in the Bleo group were dominated by Timp1+ FBs with high expression of fibrotic markers, such as *Timp1* (20), *Sparc* (21), and *Postn* (22), while the fibroblasts in the control group were mainly composed of Timp3+ FBs with highly expressed *Timp3* (23) and anti-oxidative stress markers *Gstm1* (24) and *Gsta3* (25) (**Figure 1D**).

### Polarity of fibroblasts in mouse model of pulmonary fibrosis

**Figure 2:**
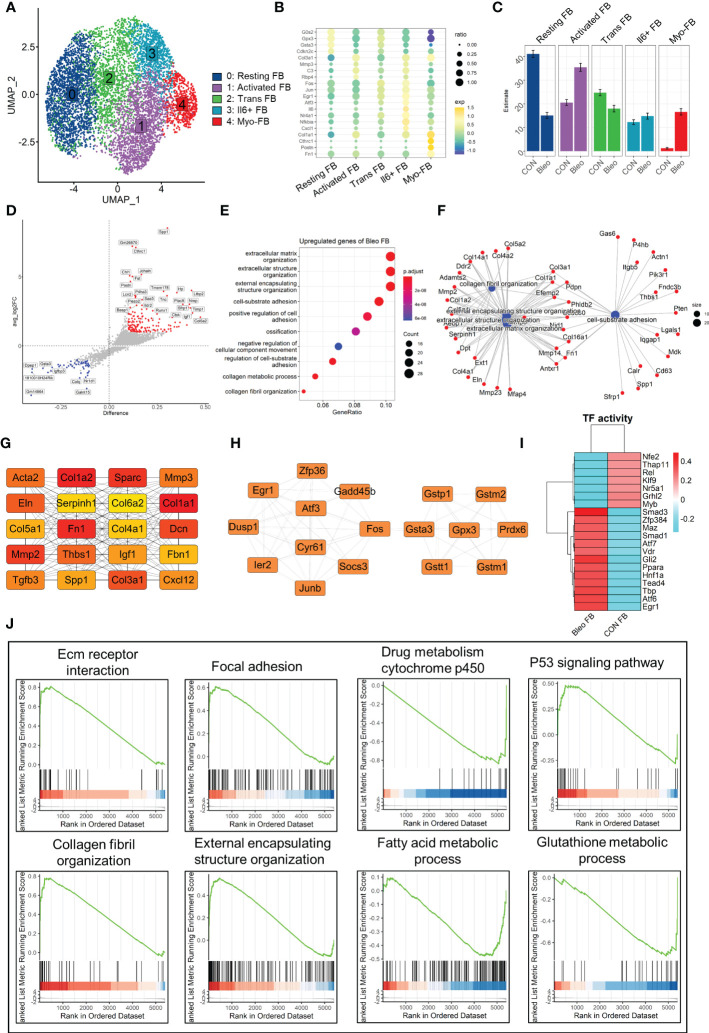
Differential characteristics of fibroblasts in Bleo and control groups. **(A)** UMAP color-coded by annotated cell fibroblast subset. **(B)** Representative markers of each cell subset. **(C)** Proportion of different cell subsets in each group color-coded by cell subset. **(D)** Volcano plot of differential genes in Bleo group relative to control group; blue dot represents significantly down-regulated gene (log2fc < -1, difference < -0.1), and red dot represents significantly up-regulated gene (log2fc > 1, difference < -0.1). **(E)** GO analysis of significantly upregulated genes in Bleo group. **(F)** Gene-pathway plot of significantly up-regulated genes in Bleo group. **(G, H)** Hub gene network of significantly up- and down-regulated genes in Bleo group. **(I)** Transcription factor activity via DoRothEA. **(J)** GSEA analysis of DEGs (Bleo vs. CON, min.pct = 0.1, logfc.threshold = 0).

Since fibroblasts are the core factor in fibrosis progression, the present study focused on fibroblast lineage and discussed the differences between the two groups. Consistently, fibroblast lineage was first extracted and the data were qualified by limiting the parameters to 200 < nfeatures < 4000 and percent.mito < 5% to obtain 7,015 high-quality cells. After dimensionality reduction clustering, a total of five cell clusters were generated and labeled 0–4 (**Figure 2A**). Cluster 0 was defined as resting fibroblasts (Resting FBs) because of the high expression of cell cycle suppressor genes *G0s2* (26) and *Cdkn2c* (27) and the expression of antioxidant stress-related genes *Gpx3* (28) and *Gsta3*. Cluster 4 highly expressed *Postn*, *Cthrc1*, *Fn1*, and *Col1a1*, which are recognized markers of fibrosis (29, 30), and was named myofibroblasts (Myo-FBs). The expression of the above fibrosis marker genes in cluster 1 was not significant, but the high expressions of *Mmp3* (31), *Rbp4* (32), *Col3a1*, and *C3* (33) in cluster 1 could promote fibrosis progress, so cluster 1 was named activated fibroblasts (Activated FBs). Cluster 2 highly expressed stress-related transcription factors, such as *Egr1* (34), *Fos*, and *Jun* (35), and also showed a gene expression pattern of resting FBs. Therefore, cluster 2 was regarded as transitional fibroblasts (Trans FBs). Finally, cluster 3 was named Il6+fibroblasts (Il6+FBs) because of its high expression of inflammatory markers *Il6* (36), *Nfkbia* (37), *Nr4a1* (38), and *Cxcl1* (39) (**Figure 2B**). Comparison of the proportions of these cell subsets between the two groups demonstrated that Resting FBs were dominant in the control group, while Activated FBs and Myo-FBs were significantly up-regulated in the Bleo group (**Figure 2C**).

Next, DEGs were evaluated between the two groups (**Figure 2D**). *Cthrc1*, *Postn*, and *Timp1* were considered to be markers of fibrosis and were significantly up-regulated in the Bleo group. The control group showed high expression of *Gsta3* and *Gstm1*, which participate in the inhibition of oxidative stress, as mentioned above. In addition, *Dpep1* was also significantly up-regulated in the control group. It has been reported to be related to ferroptosis, but has not been studied in fibrosis. With the parameters of min.pct = 0.25 and log FC.threshold = 0.25, 309 genes up-regulated in the Bleo group were screened out. These genes were significantly enriched in extracellular matrix organization, positive regulation of cell adhesion, collagen fiber organization, and other items related to fibrosis (**Figure 2E**). **Figure 2F** shows the gene network of the three most gene-enriched items. In addition, the hub gene network of up-regulated genes was analyzed in both groups using Cytoscape software. In the hub gene network of the Bleo group, *Fn1*, *Sparc*, *Acta2*, *Col1a1*, *Mmp2*, and *Mmp3* were well-recognized markers of fibrosis (**Figure 2G**). In the hub gene network of the control group, *Gstm1*, *Gsta3*, *Gpx3*, and *Gstt1* have been reported to participate in glutamine metabolism and to also play an important role in antioxidant stress (**Figure 2H**).

Then, we evaluated the transcription factor activity of the two groups *via* DoRothEA (**Figure 2I**). The results indicated that *Smad3* was the most active transcription factor in the Bleo group, Similar results were obtained by GSEA analysis, where the Bleo group was significantly enriched in fibrosis-related as well as P53 signaling pathways. suggesting that polarity of fibroblasts is involoved in pulmonary fibrosis. In addition, the fatty acid and glutathione metabolic processes were down-regulated in the Bleo group compared to the control group (**Figure 2J**).

### Differentiation of fibroblasts in bleomycin-induced pulmonary fibrosis model

**Figure 3:**
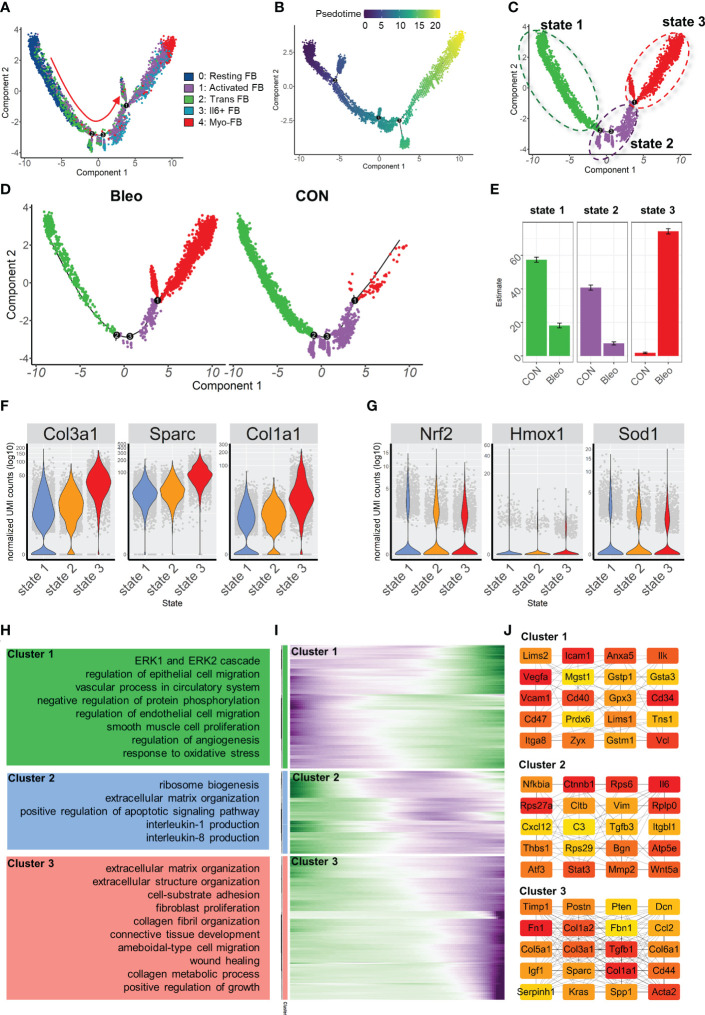
Characterization of pulmonary fibroblast development trajectory in the mouse model. **(A)** Trajectory inference analysis of fibroblast subsets color-coded by annotated cell subset. **(B)** Trajectory color-coded by pseudotime. **(C)** Trajectory color-coded by cell state. **(D)** Trajectories of two groups color-coded by cell state. **(E)** Proportion of cell state in two groups color-coded by cell state. **(F, G)** Expression levels of fibrosis- and antioxidant stress-related genes in each cell state. **(H)** GO analysis for genes in each cluster. **(I)** Gene expression dynamics during differentiation of state 1 to state 3. **(J)** Top 20 hub gene networks in each cluster ranked by ‘degree’.

In order to further explore the mechanism of phenotypic changes in fibroblasts, pseudotime analysis was performed to infer the development trajectory of fibroblasts *via* Monocle. In this cell trajectory, Resting FBs were the starting point of the trajectory, transitioning from Trans FBs to Activated FBs and finally ending up as Myo-FBs (**Figures 3A, B**). The whole trajectory was divided into three segments based on branch points 1 and 2 and named states 1, 2, and 3 according to the developmental order (**Figure 3C**). In the trajectory, a higher proportion of state 3, which is the end stage of differentiation, was found in the Bleo group compared to the control group. In contrast, the proportions of state 1, which is the starting point of the trajectory, and intermediate state 2 were lower than those in the control group (**Figures 3D, E**). A selection of fibrosis marker genes and their expression levels were progressively upregulated in these three cell states, while the marker genes associated with resistance to oxidative stress were progressively down-regulated (**Figures 3F, G**). Finally, analysis of the genetic dynamics of the trajectories showed that these genes aggregated into three clusters. The expression levels of genes in cluster 1 were progressively down-regulated with the developmental trajectory. GO analysis showed that genes in cluster 1 were mainly enriched in BPs of angiogenesis and response to oxidative stress. The expression levels of genes in clusters 2 and 3 were progressively up-regulated with developmental trajectory. These genes were mainly enriched in the biological programs of inflammatory response and fibrosis (**Figures 3H, I**). **Figure 3J** shows the top 20 hub gene networks in each cluster.

### Cuproptosis levels were negatively correlated with fibrosis levels in the lung fibrosis model

**Figure 4:**
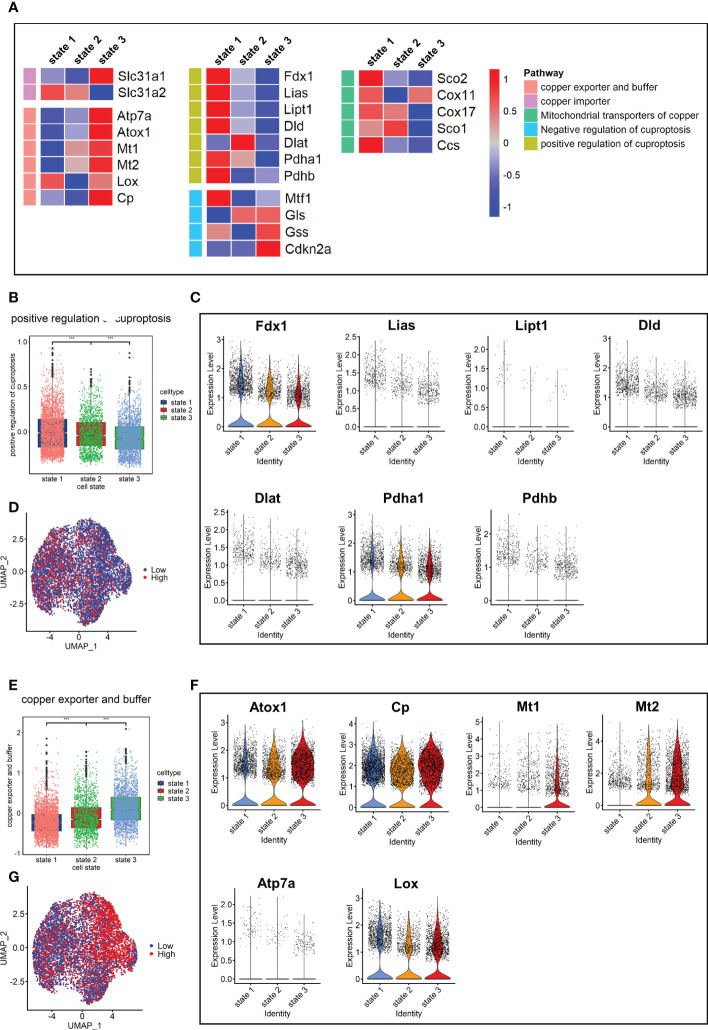
Characterization of copper ion metabolism and cuproptosis level in mouse pulmonary fibroblasts. **(A)** Heatmap of genes related to copper ion metabolism and cuproptosis. **(B)** Scores of gene sets related to positive regulation of cuproptosis. **(C)** Expression level of genes related to positive regulation of cuproptosis. **(D)** UMAP color-coded by level of gene sets promoting cuproptosis. **(E)** Scores of gene sets related to copper export and buffering. **(F)** Expression level of genes related to copper export and buffering. **(G)** UMAP color-coded by level of gene sets related to copper export and buffering.

Since cuproptosis has not been studied in pulmonary fibrosis, the expression of cuproptosis-promoting genes *Fdx1*, *Pdhb*, *Pdha1*, *Dld*, *Dlat*, *Lias*, and *Lipt1* (13) were evaluated in fibroblasts. The results showed that the expression levels of these genes in the Bleo group were all lower than those in the control group (**Supplementary Figure 1A**). We also evaluated the expression of these genes in each cell state. The results showed that the genes promoting cuproptosis were highly expressed in state 1, which was composed of resting fibroblasts, but had a low expression in state 3, which was composed of cells with high fibrotic levels. However, the expression level of genes related to cuproptosis inhibition showed the opposite trend (**Figure 4A**). The expression level of genes related to copper ion export and buffering gradually increased in the order of cell state. These gene sets were scored through the AddModuleScore function, showing that the gene sets related to cuproptosis promotion were down-regulated with the development of cell state. The expression level of these genes in each cell state was consistent with it (**Figures 4B-D**). With the development of cell state, the gene sets related to copper ion export and buffering were gradually up-regulated, especially *Atox1*, *Mt1*, and *Mt2* (**Figures 4E-G**). It has been reported that cuproptosis is closely related to the tricarboxylic acid cycle. Therefore, the key enzymes or genes of five metabolic pathways were evaluated in each cell state, including pentose phosphate pathway, glycolysis, tricarboxylic acid cycle, mitochondrial metabolism, and lipid metabolism. The results showed that the metabolic level of TCA cycle in state 1 was the highest, followed by states 2 and 3. The lipid metabolism level decreased gradually, while mitochondrial metabolism level tended to increase with cell state (**Supplementary Figure 1B**).

### Single-cell atlas of human pulmonary fibrosis

Next, the investigation focused on the scRNA-seq data for human pulmonary fibrosis to further explore its relationship with cuproptosis. Data for human pulmonary fibrosis provided by Tsukui et al. included three samples of normal lung tissue (Normal), three samples of idiopathic pulmonary fibrosis (IPF), and two samples of scleroderma (SCD).

**Figure 5:**
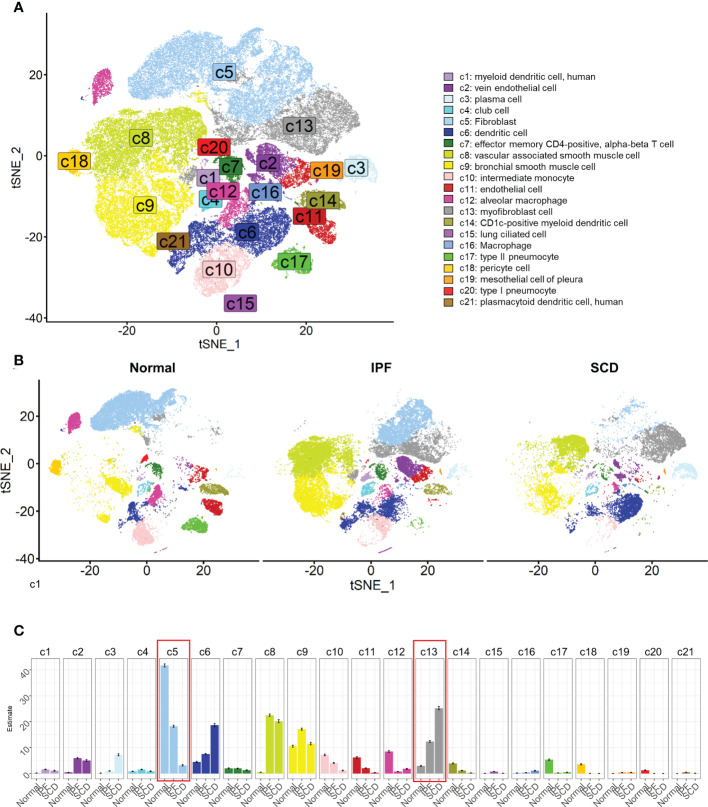
Cell lineages of normal and diseased lung tissues in humans. **(A)** t-SNE color-coded by annotated cell lineage. **(B)** t-SNE displayed according to groups and color-coded by cell lineage. **(C)** Proportion of different cell lineages in each group color-coded by cell lineage.

After quality control, a total of 79,907 cells were obtained and dimension reduction and clustering were performed. Then, the scRNA-seq data for human lung tissue provided by Travaglini et al. (40) were used as the reference data set. The data were automatically annotated using singleR and a total of 21 cell lineages were identified (**Figure 5A**). Consistent with the mouse model, there was a great difference between fibroblasts in normal and fibrotic lung tissue. The fibroblasts were mainly composed of myofibroblasts in IPF and SCD. Compared to the mouse pulmonary fibrosis model, various cell lineages in human lung tissue had significant polarity among different groups, such as vascular-associated smooth muscle cells, dendritic cells, and alveolar macrophages (**Figures 5B, C**). These results indicated that the inflammatory response also plays an important role in the process of human pulmonary fibrosis.

### Heterogeneity of human pulmonary fibroblasts

**Figure 6:**
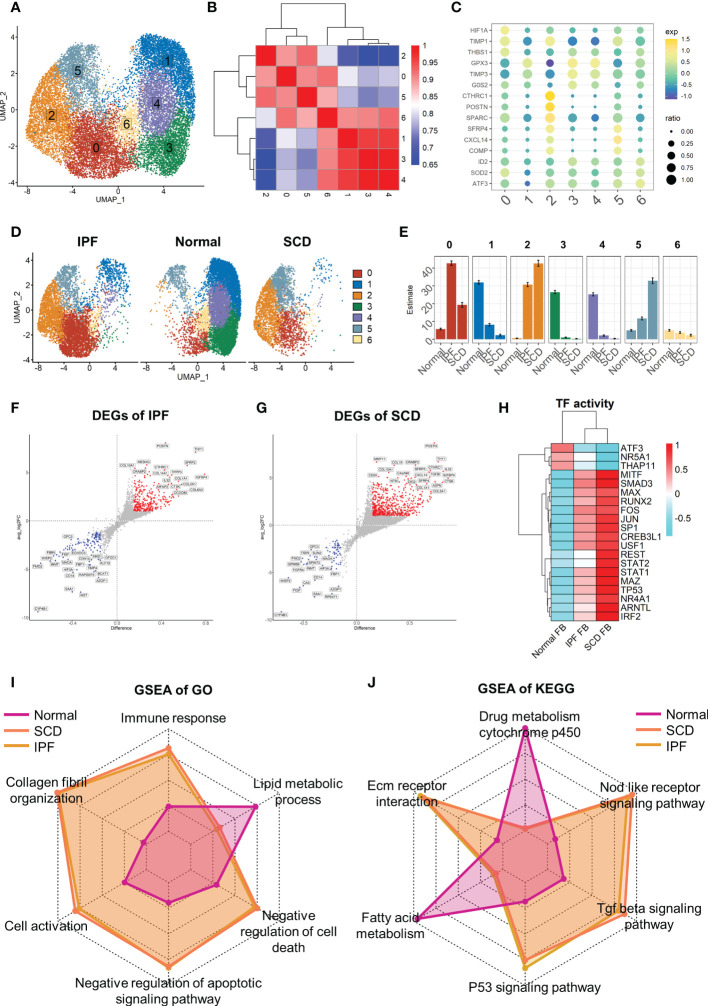
Full characterization of differences in lung fibroblasts between disease and normal groups. **(A)** UMAP color-coded by cell subset. **(B)** Cluster map of top 2000 markers of each cell subsets. **(C)** Representative markers of each cell subset. **(D)** t-SNE of two groups color-coded by cell subset. **(E)** Proportion of different cell subsets in each group color-coded by cell subset. **(F, G)** Volcano plot of differential genes in IPF and SCD groups relative to Normal group. **(H)** Transcription factor activity determined via DoRothEA. **(I, J)** GSEA analysis of Normal, IPF, and SCD groups displayed by radar plot and color-coded by group.

In-depth study of fibroblast heterogeneity will help to understand the mechanism of pulmonary fibrosis. Therefore, fibroblast lineage was selected for further analysis. A total of 18,197 fibroblasts were screened out and seven fibroblast subsets were identified and labeled 0–6 (**Figure 6A**). These subsets were clustered with the top 2000 marker genes in each subset. The results showed that subsets 1, 3, and 4 and subsets 0, 2, and 5 were clustered together, suggesting that they have unique gene expression patterns (**Figure 6B**). Interestingly, fibroblasts showed obvious polarity in the disease and normal groups. Subsets 1, 3, and 4 constituted the majority of fibroblasts in normal lung tissue, while subgroups 0, 2, and 5 dominated in IPF and SCD (**Figure 6D, E**). Subsets 1, 3, and 4 highly expressed *GPX3*, *TIMP3*, and *G0S2*, suggesting a phenotype of low proliferation, low fibrosis, and high antioxidant stress. Subset 0 strongly expressed *HFIA*, *TIMP1*, and *THBS1*, all of which are associated with fibrosis (41, 42). Subset 2 was considered highly fibrotic due to high expression of *POSTN*, *CTHRC1*, and *SPARC* (**Figure 6C**).

Taking the healthy group as the control, differential gene analysis of IPF and SCD represented them on a volcano plot (**Figures 6F, G**). Up-regulated genes in IPF and SCD, such as *POSTN*, *CTHRC*, and *COL3A1*, were involved in promoting fibrosis. Interestingly, a recognized marker of anti-pulmonary fibrosis *THY1* (43) was highly expressed in IPF and SCD, which may be related to the mechanism of post-transcriptional regulation (**Figure 6H**). Analysis of transcription factor activity suggested that *SMAD3* and *MITF* were the most active transcription factors in IPF and SCD (**Figure 6I**). Functional differences among Normal, IPF, and SCD groups were then analyzed by GSEA and displayed on a radar plot. This result was consistent with animal study outcomes showing that IPF and SCD had a higher resistance to apoptosis and cell death, lower levels of lipid metabolism, and greater number of fibrosis-related BPs (**Figure 6J**).

### Differentiation trajectory of fibroblasts in human pulmonary fibrosis

**Figure 7:**
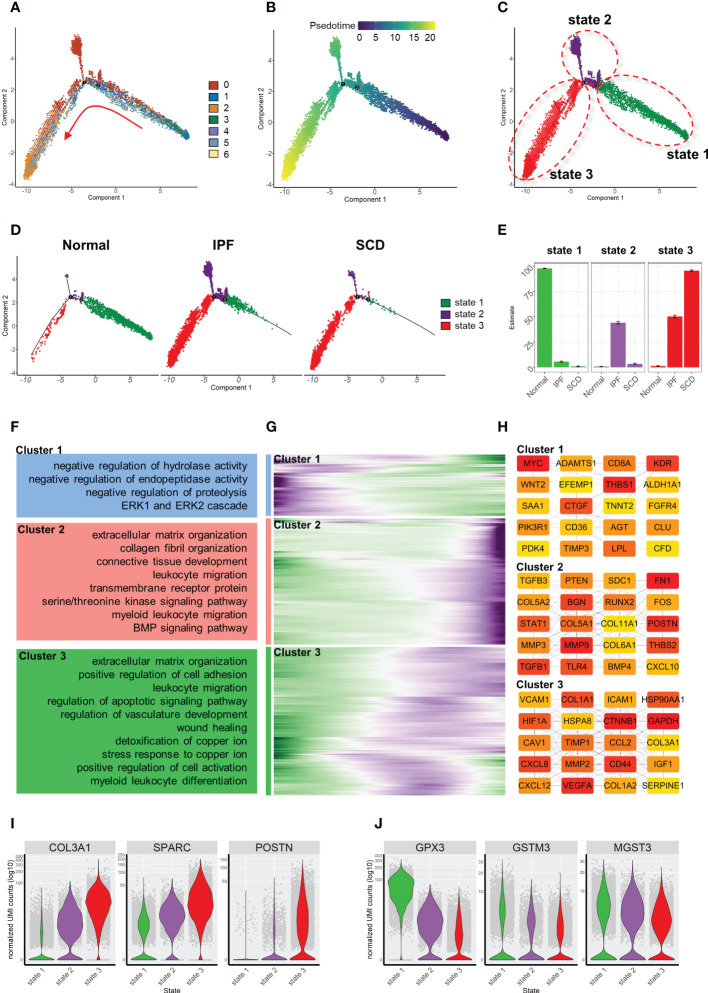
Characterization of human pulmonary fibroblast development trajectory. **(A)** Trajectory inference analysis of fibroblasts color-coded by cell subset. **(B)** Trajectory color-coded by pseudotime. **(C)** Trajectory color-coded by cell state. **(D)** Normal, IPF, and SCD trajectories color-coded by cell state. **(E)** Proportion of cell state in three groups color-coded by cell state. **(F)** GO analysis of genes in each cluster. **(G)** Gene expression dynamics during differentiation of state 1 to state 3. **(H)** Top 20 hub gene networks in each cluster ranked by ‘degree’. **(I, J)** Expression levels of fibrosis- and antioxidant stress-related genes in each cell state.

Similarly, the fibroblast trajectory was inferred using Monocle and divided into three segments: states 1, 2, and 3 (**Figures 7A-C**). State 1 was the starting point of the trajectory and was mainly composed of subsets 1, 3, and 4. State 2 was composed of subset 2. State 3 was the trajectory end-point and mainly composed of subsets 2 and 5. Consistent with animal study results, the normal group was dominated by state 1, while IPF and SCD were dominated by states 2 and 3 (**Figures 7D, E**). Gene dynamic analysis was used to analyze the changes in gene expression patterns and functions during fibroblast differentiation. These differential genes were divided into three clusters. Fibrosis and inflammatory reactions were gradually up-regulated with differentiation, which was consistent with animal study results. Notably, the genes in cluster 3 were enriched in copper ion detoxification and stress response to copper ion (**Figures 7F-H**). In addition, *COL3A1*, *SPARC*, and *POSTN* gradually increased, while *GPX3*, *GSTM3*, and *MGST3* gradually decreased with the order of cell state, suggesting that fibrosis was aggravated progressively and was accompanied by anti-oxidative stress ability damage in fibroblast differentiation (**Figures 7I, J**).

### Cuproptosis levels were negatively correlated with fibrosis levels in human lung fibrosis

**Figure 8:**
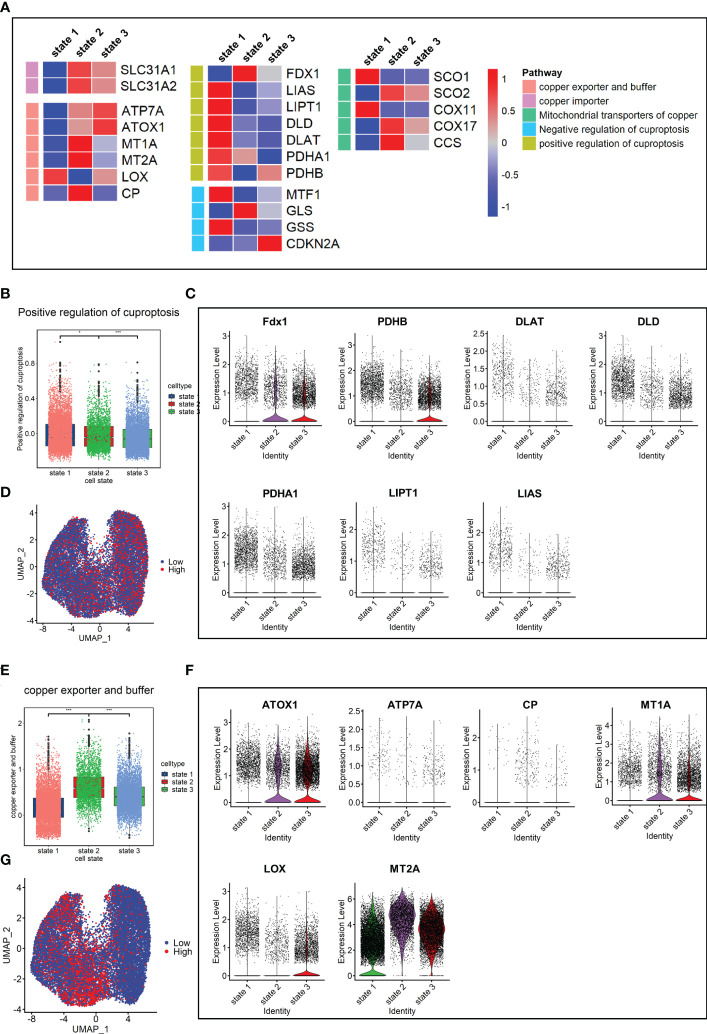
Copper ion metabolism and cuproptosis level in mouse pulmonary fibroblasts. **(A)** Heatmap of genes related to copper ion metabolism and cuproptosis in cell states. **(B)** Scores of gene sets related to cuproptosis promotion. **(C)** Expression level of genes related to cuproptosis promotion. **(D)** UMAP color-coded by levels of gene sets related to cuproptosis promotion. **(E)** Scores of gene sets related to copper export and buffering. **(F)** Expression level of genes related to copper export and buffering. **(G)** UMAP color-coded by level of gene sets related to copper export and buffering.

Finally, the expression levels of genes related to copper metabolism and energy metabolism were determined in fibroblasts. The results showed that the expression levels of seven cuproptosis-promoting genes in the fibrosis group (IPF+SCD) were all lower than those in the normal group (**Supplementary Figure 1C**). In terms of the order of cell state, the fibroblasts in state 1 had a higher expression level of genes related to cuproptosis promotion and lower expression level of genes related to copper export and buffering (**Figure 8A**). The cuproptosis scores and expression levels of genes related to cuproptosis decreased with the order of cell state, which was consistent with results in animal experiments (**Figures 8B-D**). In human data, the gene expression level related to copper ion buffering was higher in state 2, while in the animal data, it was higher in state 3 (**Figures 8E-G**). In addition, the levels of lipid metabolism and tricarboxylic acid cycle metabolism gradually decreased with the order of cell state, while the level of mitochondrial metabolism gradually increased, which was consistent with animal study results (**Supplementary Figure 1D**).

## Discussion

The present study investigated the relationship between genes related to cuproptosis and pulmonary fibrosis based on the scRNA-seq data from animal models and human specimens. These genes maintained a certain level of expression in the healthy control group, but were significantly down-regulated in the fibrosis group. The expression level of genes related to cuproptosis gradually decreased with the differentiation of fibroblasts from the resting state to myofibroblasts. In addition, the process of cell differentiation was accompanied by metabolic reprogramming, and the levels of tricarboxylic acid cycle and lipid metabolism gradually decreased, while the level of mitochondrial metabolism gradually increased.

To the best of our knowledge, cuproptosis has been investigated in the field of tumor research. The present study is the first to explore the role of cuproptosis in fibrosis. Surprisingly, a consistent phenotype was observed in animal and human studies on pulmonary fibrosis, where the level of cuproptosis was negatively correlated with the level of fibrosis, suggesting a potential role in pulmonary fibrosis.

As a cofactor of a variety of enzymes, copper ion participates in catalytic reactions and is an indispensable microelement in the body (44). Intracellular copper overload is closely related to poor prognosis, such as in Wilson’s disease that is caused by ATP7B mutation (45). Previous studies have shown that elevated copper ion levels in some cancers are associated with poor prognosis (46). It may also be involved in promoting angiogenesis through the copper-HIF1A-VEGF pathway (47). Copper binding protein SPARC (48) is involved in tumor invasion (49) and controlling copper ion levels by copper chelating agents seems to be a feasible method for tumor treatment (50). Tumors are characterized by uncontrollable cell proliferation. Cuproptosis is a newly discovered form of programmed cell death that provides a new perspective for anti-tumor treatment. In addition,some fibrotic diseases may also be regulated by the level of copper ions, partly because the elevated intracellular copper ions activate lysyl oxidase to enhance the cross-linking of collagen and elastin (51, 52). However, some studies reported that copper ions may be involved in alleviating fibrosis in liver and heart, indicating the effects of copper ions on fibrosis in different tissues are heterogeneous (53, 54),. In fact, excessive proliferation and activation of fibroblasts is one of the characteristics of fibrotic diseases. Studies have shown that the anti-apoptotic ability of fibroblasts may be involved in promoting the progression of fibrosis (9). Promoting apoptosis may thus be an effective solution to fibrosis (55, 56). In the present study, fibroblasts in IPF and SCD had the ability to negatively regulate apoptosis and cell death (**Figure 6I, J**). In normal mice and healthy humans under physiological conditions, lung fibroblasts exhibited a certain level of cuproptosis, which gradually decreased with the differentiation of fibroblasts to myofibroblasts with a high fibrotic phenotype. It has been suggested that an appropriate cuproptosis level may play the same role as apoptosis in limiting excessive fibroblast proliferation and activation.

However, the mechanism of decreasing the level of cuproptosis remains unclear. According to the available data, genes related to copper ion export and buffering were at a low level under physiological conditions, which may maintain intracellular copper ion concentration, thus inducing a certain level of cuproptosis. With the activation of fibroblasts, the expression level of genes related to copper ion export and buffering gradually increased, which may reduce the concentration of copper ions, thus inhibiting the induction of cuproptosis. In fact, this mechanism may be even more complicated. If copper ion is the only factor inducing cuproptosis, it would conflict with the fibrosis improvement by copper chelate therapy that has been reported previously (57).

It is reported that cuproptosis is more sensitive to cells with high level of TCA cycle, with copper ions binding to the lipid acylation components of TCA cycle to induce toxic protein stress (13). In this study, we found that the expression levels of key genes related to the TCA cycle were consistent with genes involved in cuproptosis, which is also consistent with literature. Metabolic reprogramming is one of the characteristics of fibrosis (58). We observed that the metabolic level of TCA cycle in fibroblasts decreased with the progression of fibrosis, which may be responsible for the reduced level of cuproptosis.

There is a limitation needed to be pointed out. The present study concluded the negative correlation between cuproptosis in fibroblasts and pulmonary fibrosis using only bioinformatics tools. Thus the causal relationship between cuproptosis and pulmonary fibrosis have not been clarified. Therefore, future research should be undertaken to explore the effects of up-regulation or down-regulation of cuproptosis on pulmonary fibrosis.

## Data availability statement

The datasets presented in this study can be found in online repositories. The names of the repository/repositories and accession number(s) can be found in the article/**Supplementary Material**.

## Author contributions

GL and GXL developed the main research plan. GXL and LHP analyzed the data, generated charts, and wrote the manuscript. ZC, MJW and YPZ helped to collect data and assemble references. All authors contributed to the article and approved the submitted version.
