# Supplementary material for: Appropriate level of cuproptosis may be involved in alleviating pulmonary fibrosis
Source: Front Immunol. 2022 Dec 19;13:1039510. doi: 10.3389/fimmu.2022.1039510 (PMC9806118; doi:10.3389/fimmu.2022.1039510)
Supplement: Supplementary file 1 [file DataSheet_1.docx]

**Supplementary Figure 1**


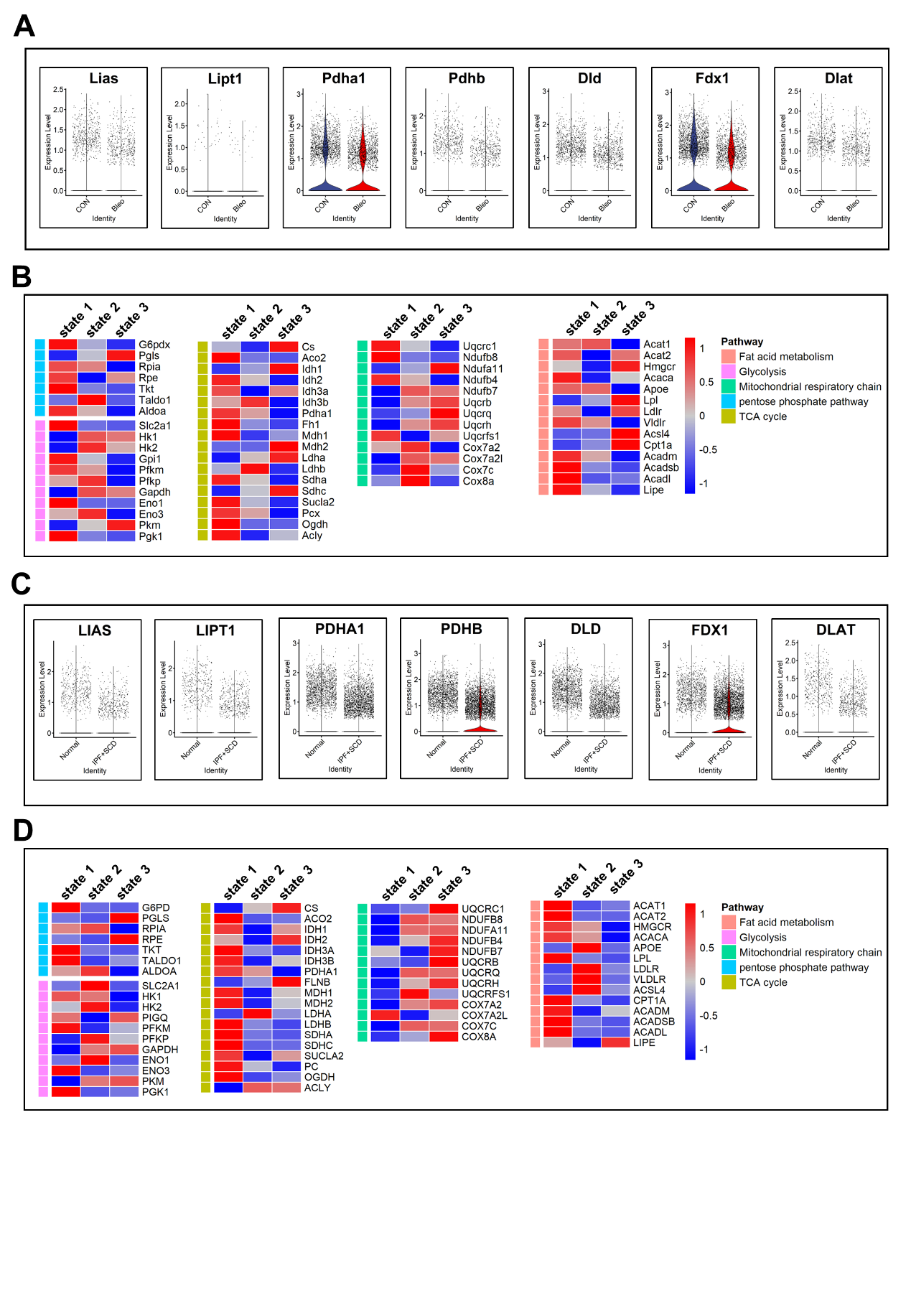
 Evaluation of cuproptosis and metabolism in mouse and human. (A) Expression level of cuproptosis promotion-related genes in Bleo and CON groups. (B) Heatmap of metabolic levels of fibroblasts in mice. (C) Expression level of genes related to cuproptosis promotion in normal and fibrotic (IPF+SCD) groups. (D) Heatmap of metabolic levels of human fibroblasts.
